# The past and future of peri-operative interventions to reduce arthrogenic quadriceps muscle inhibition after total knee arthroplasty: A narrative review

**DOI:** 10.1016/j.ocarto.2023.100429

**Published:** 2023-12-21

**Authors:** Laura Churchill, Michael John Bade, Ryan C. Koonce, Jennifer E. Stevens-Lapsley, Thomas Bandholm

**Affiliations:** a Physical Therapy Program, Department of Physical Medicine and Rehabilitation, University of Colorado Anschutz Medical Campus, Aurora, CO, USA; b Department of Orthopaedic Surgery, University of Colorado School of Medicine, Highlands Ranch, CO, USA; c Eastern Colorado VA Geriatric Research Education and Clinical Center (GRECC), Aurora, CO, USA; d Physical Medicine & Rehabilitation Research-Copenhagen (PMR-C), Department of Physical and Occupational Therapy, Copenhagen University Hospital Amager-Hvidovre, Hvidovre, Denmark; e Department of Clinical Research, Copenhagen University Hospital Amager-Hvidovre, Hvidovre, Denmark; f Department of Orthopedic Surgery, Copenhagen University Hospital Amager-Hvidovre, Hvidovre, Denmark

**Keywords:** Arthroplasty, Knee replacement, Muscle inhibition, Arthrogenic, Rehabilitation

## Abstract

Total knee arthroplasty (TKA) improves patient-reported function by alleviating joint pain, however the surgical trauma exacerbates already impaired muscle function, which leads to further muscle weakness and disability after surgery. This early postoperative strength loss indicates a massive neural inhibition and is primarily driven by a deficit in quadriceps muscle activation, a process known as arthrogenic muscle inhibition (AMI). To enhance acute recovery of quadriceps muscle function and long-term rehabilitation of individuals after TKA, AMI must be significantly reduced in the early post-operative period. The aim of this narrative review is to review and discuss previous efforts to mitigate AMI after TKA and to suggest new approaches and interventions for future efficacy evaluation. Several strategies have been explored to reduce the degree of post-operative quadriceps AMI and improve strength recovery after TKA by targeting post-operative swelling and inflammation or changing neural discharge. A challenge of this work is the ability to directly measure AMI and relevant contributing factors. For this review we focused on interventions that aimed to reduce post-operative swelling or improve knee extension strength or quadriceps muscle activation measured by twitch interpolation. For individuals undergoing TKA, the use of anti-inflammatory medications, tranexamic acid, cryotherapy, intra-articular drains, torniquets, and minimally invasive surgical techniques for TKA have limited benefit in attenuating quadriceps AMI early after surgery. However, interventions such as inelastic compression garments, voluntary muscle contractions, and neuro-muscular electrical stimulation show promise in mitigating or circumventing AMI and should continue to be refined and explored.

## 1 Introduction

Total knee arthroplasty (TKA) is commonly performed to relieve pain and improve function among individuals with severe knee osteoarthritis. While pain relief is often achieved after surgery over the course of several months [1], the surgical trauma exacerbates the underlying muscle weakness and disability from OA, leading to long-term atrophy and further disability.

Enhanced Recovery After Surgery (ERAS) programs for individuals undergoing TKA have been developed to optimize patient recovery and resolve issues that lead to complications via a multi-modal, multidisciplinary, and evidence-based approach [2]. Such programs have demonstrated enhanced recovery after surgery, with decreased need for hospitalization, convalescence, and risk of medical complications [3]. Despite these efforts and documented improvements, ERAS protocols fail to substantially modify the pathophysiological stress response to the surgical trauma. As a result, patients lose 60–83 ​% knee-extension strength on average in the operated leg during the initial post-operative period [4,5]. This early postoperative strength loss indicates a massive neural inhibition and is primarily driven by a deficit in quadriceps muscle activation [5], a process known as arthrogenic muscle inhibition (AMI) [6].

In the early post-operative period, quadriceps AMI poses challenges for patients in navigating their home environment and recovering basic function which contributes to an increase risk of falls in the early post-operative period [7]. Long-term, quadriceps AMI contributes to muscle atrophy and can delay or prevent effective strengthening and rehabilitation [6]. Indeed, lower extremity muscle weakness persists many years after TKA with chronic quadriceps strength deficits of up to 42 ​% observed compared to healthy age-matched controls [8]. The consequences of this are profound, especially among older adults, as quadriceps muscle weakness is associated with decreased gait speed, balance, ability to climb stairs and rise from a chair, and increases risk of falls [[9], [10], [11], [12], [13], [14], [15]]. To enhance acute recovery of quadriceps muscle function and long-term rehabilitation of individuals after TKA, AMI must be significantly reduced in the early post-operative period [16].

The aim of this narrative review is to introduce AMI, discuss previous efforts to mitigate AMI or clinical surrogate measures of AMI after TKA, and to suggest new approaches and interventions for future efficacy evaluation.

### 1.1 What is AMI

AMI is defined as “a presynaptic, ongoing reflex inhibition of musculature surrounding a joint after distension or damage to structures of that joint” and is characterized by a diminished ability to contract the quadriceps musculature after TKA [17]. It is generally accepted that AMI is caused by a change in the discharge of articular sensory receptors from the damaged knee joint, which affects the central nervous system by changing the excitability of multiple spinal and supraspinal pathways [6]. The combined effect is reduced quadriceps muscle activation [6] which impacts knee-extension strength. The change in joint afferent discharge likely originates from a combination of local swelling, inflammation, joint laxity, and damage to joint afferents [6] – factors that are well known clinically after TKA. This disruption of afferent input leads to reflexive inhibition at a spinal cord level decreasing the excitability of alpha motor neurons [18]. This change in afferent input in response to damage or distension of the joint is thought to be the most influential factor associated with acute AMI [17,19]. However acute changes in afferent input also ascend to higher brain regions, which over time, induce a variety of neuroplastic adaptions impacting the motor cortex. These changes influence how the motor cortex sends descending signals, further compounding decreased activation of alpha motor neurons, and resultant muscle contractions [18].

### 1.2 How is AMI measured

Since AMI is a reduction in motor neuron pool excitability (i.e., fewer motor neurons to recruit) and central activation failure (i.e. diminished ability to voluntary recruit motor neurons), it can be quantified by measuring voluntary force of the motor neuron pool [17,19]. Direct measures of AMI that are commonly used in research settings include the interpolated twitch technique, or burst superimposition [6]. Indirect measures of AMI include measuring voluntary force output of the quadriceps via dynamometry or a clinical battery of quadriceps activation [17,20].

The interpolated twitch technique and burst superimposition involve electrically stimulating the quadriceps using surface electrodes placed on the thigh muscle during a maximum voluntary contraction [6]. By superimposing an evoked stimulation on a voluntary contraction, muscle fibers that are not activated by the central nervous system are activated, producing additional muscle force, and revealing incomplete voluntary activation. Any additional muscle force generated by the superimposed stimulation has a linear relationship to the initial voluntary force [21]. For the interpolated twitch method, activation failure is calculated via the formula: 1 - (superimposed twitch force at maximum effort/superimposed twitch force at rest) x100, where a value of 100 ​% represents full voluntary activation, whereas values less than 100 ​% indicate incomplete activation [6,20,22]. For burst superimposition, voluntary activation is calculated via the formula: maximum effort torque/(maximum effort torque ​+ ​superimposed stimulus torque) [6]. This calculation produces a central activation ratio where a value of 1.0 indicates complete activation and values ​< ​1.0 indicate incomplete activation [23].

Indirect measures of AMI include measuring voluntary force output [17] (i.e., muscle strength testing) which can be measured via electromechanical or handheld dynamometry [24]. After TKA, comparison of maximum voluntary contraction force of the quadriceps using dynamometry pre-operatively and again shortly after surgery offers a way to indirectly quantify the degree of AMI, which has been argued to be most severe in the acute stages after joint damage [6]. Another easy way to implement a surrogate measure of AMI is a clinical quadriceps activation battery which includes the patient performing 1) isometric quadriceps contraction, 2) straight leg raise, and 3) the quadriceps extension lag test, where each test is scored on a 3-pt scale from 0 ​= ​unable to perform, to 2 ​= ​able to perform fully. The scores from the 3 tests are summed (range 0–6) with higher scores indicating better performance. Bade et al. investigated the relationship between this clinical quadriceps activation battery performed 4 days after TKA and the twitch interpolation measure of quadriceps activation 1-month after TKA and found that lower scores in the clinical battery (<3/6) were correlated to poorer quadriceps activation [20].

Another important measure related to AMI is knee joint swelling. Interventions that can manipulate knee joint fluid could theoretically change quadriceps AMI and knee-extension strength shortly following TKA. The link between swelling and AMI has been shown mainly in pre-post analyses in studies that have experimentally induced effusion into otherwise healthy knee joints and saw immediate decreases in quadriceps strength [[25], [26], [27], [28], [29]]. More data is needed to support a causal relationship between reducing clinical knee swelling and improved quadriceps activation. This could be achieved by aspirating fluid from the clinically swollen knee joint (arthrocentesis) and measuring activation and knee strength immediately after this procedure. This would provide proof of principle for a causal link in clinical knee swelling, and it would align data from “antagonistic” knee swelling models where quadriceps muscle activation is acutely reduced when fluid is injected into a healthy human knee joint [[25], [26], [27], [28], [29]].

### 1.3 Timeline of AMI/Quadriceps strength recovery after TKA

Some degree of quadriceps activation failure is normal (∼5 ​% or a CAR ​= ​0.95) and exists in healthy subjects when measured by burst superimposition [30,31]. After TKA, quadriceps activation failure is marked, and this AMI is a significant driver of quadriceps strength loss after surgery [5,32]. Specifically, when examining the relative contributions of activation failure and cross-sectional muscle area to quadriceps strength losses after TKA, failure of voluntary activation accounts for nearly twice the amount of quadriceps strength loss compared to quadriceps atrophy at 1-month post-operatively [5]. Thus, quadriceps strength loss shortly after TKA represents a meaningful estimate of AMI.

A recent systematic review and meta-analysis of 17 studies (n ​= ​832 patients) explored the magnitude of quadriceps strength loss and timing of quadriceps strength recovery after TKA. In general, they found that quadriceps strength declines markedly in the immediate post-operative period and gradually and linearly increases over time up to 6-months post-operatively. Compared to pre-operative values, the greatest observed decline in quadriceps strength was observed at 3-days post-operatively, remained significantly lower at 3-months post-operatively, and was not fully recovered at 6-months post-operatively. In the longer-term at 12-months post-operatively, quadriceps strength was significantly recovered compared to pre-operative values and plateaued at 2-years post-operatively [33].

Overall, few studies have directly measured or diagnosed AMI in the acute postoperative period after TKA, however it is well accepted that it is present and most severe in the acute postoperative phase [5,6,34] and contributes to strength deficits after surgery [5,34]. As such, we acknowledge that this review includes studies that use surrogate measures of AMI in patients shortly after TKA.

## 2 Past efforts to reduce AMI

Several strategies have been explored to reduce the degree of post-operative quadriceps AMI and improve strength recovery after TKA by targeting post-operative swelling and inflammation or changing neural discharge. A challenge of this work – when performed shortly following a TKA – is the ability to directly measure AMI and relevant contributing factors, such as changes in the discharge of specific afferents within the knee joint capsule (i.e., mechanoreceptors such as Ruffini endings and Pacinian corpuscles) [35]. Most prior research has used surrogate measures of AMI as they can be applied at scale shortly after surgery when AMI is pronounced, and disuse-atrophy is not. In most cases, measures of knee-extension strength are used as AMI is a significant driver of quadriceps strength loss after surgery. If an experimental procedure has a clinically relevant effect on quadriceps AMI, it should be reflected by a corresponding change in knee-extension strength shortly after surgery. In other cases, researchers have used measures such as twitch interpolation and burst superimposition to reflect quadriceps AMI more closely after TKA [[36], [37], [38], [39]]. When we discuss the different findings below, we have made efforts to clearly indicate which methodology was used to assess AMI.

### 2.1 Cryotherapy

In models of clinical knee swelling, local ice application reduces direct measures of quadriceps AMI in experimentally effused – but otherwise healthy – human knees [25,40,41]. The effect of local ice application on indirect measures of quadriceps AMI has also been observed in clinical knee swelling. Individuals with tibiofemoral knee osteoarthritis and individuals post knee ligament reconstruction or arthroscopic knee surgery have demonstrated increased knee-extension strength immediately after cryotherapy treatment [42,43]. In the TKA-population, most studies have focused on the effect of cryotherapy on pain, blood loss, and range of motion [44]. A recent systematic review suggests cryotherapy may reduce pain post-TKA, however, results are inconsistent and evidence is from low-quality studies [44]. The studies that have investigated the effect of cryotherapy on knee swelling and knee-extension strength after TKA generally indicate no effect on these measures [44,45]. Holm et al. tried to experimentally reduce afferent discharge from the operated knee joint by cooling it for 30 ​min at 7 or 10 days after TKA in a cross-over study where 24 patients were randomized to receive 30 ​min of elbow icing (control treatment) on one day and 30 ​min of knee icing on the other day (active treatment). A blinded treatment assessor measured maximal knee extension strength via a fixed handheld dynamometer 2–5 ​min before and 2–5 ​min after both treatments. They found no acute effect of knee icing on knee-extension strength (change associated with knee icing, mean ​= ​−0.01, SD ​= ​0.07 Nm/kg, change associated with elbow icing, mean ​= ​−0.02, SD ​= ​0.07 Nm/kg, P ​= ​0.493) [46]. Taken together, these results suggest that despite sound physiologic basis and preliminary evidence, cryotherapy applied for short duration (30 ​min) has limited benefit in reducing swelling or improving knee extension strength (indirect measure of AMI). However, given mixed findings, cryotherapy may have some utility for post-operative pain reduction.

### 2.2 Reducing the inflammatory response

#### 2.2.1 Drug therapies

In patients with rheumatoid arthritis, reducing post-operative inflammation by administration of corticosteroids increases quadriceps muscle activity and force output [47], an effect attributed to reduced AMI [6]. Lindberg-Larsen et al. randomized individuals undergoing TKA, to a pre-operative high-dose (125 ​mg intravenous) of methylprednisolone (n ​= ​33) or intravenous saline (n ​= ​28) and investigated the change in knee-extension strength via dynamometry, swelling via knee circumference, inflammation via plasma C-reactive protein (CRP), functional performance and pain, from baseline (preoperative) to discharge (48 ​h after surgery). The methylprednisolone group demonstrated reduced post-operative inflammation at discharge (MP Group: Median ​= ​83, IQR ​= ​56–125 CRP mg/L; Control: Median ​= ​192, IQR ​= ​147–258 CRP mg/L, p=<0.001), however, there were no observed effects on swelling, pain, functional performance, or knee-extension strength at discharge. Both groups demonstrated the typical significant loss of knee-extension strength (∼89 ​%) after surgery [48]. Based on limited evidence, it appears that corticosteroids may reduce post-operative inflammation in TKA but seem to have limited benefit in improving an indirect measure of quadriceps AMI (knee extension strength) or swelling shortly following surgery.

Tranexamic acid is another drug therapy commonly used in TKA. The primary goal of utilizing tranexamic acid is to minimize blood loss, which may contribute to reductions in lower limb swelling after TKA and thus could plausibly impact AMI. There are several methods of administration including intravenous (IV), topical, and oral as well as combinations which have proven equally effective in reducing risk of blood transfusion and blood loss [49]. Fewer studies have specifically focused on the impact of tranexamic acid on post-operative swelling and strength. Ishida et al. randomized individuals undergoing TKA to an intra-articular injection of tranexamic acid (n ​= ​50) or a saline injection (n ​= ​50). Among outcomes related to AMI they measured lower limb swelling via circumferential measurements pre-operatively and at 1-, 2-, and 4-weeks post-operative. They calculated the incremental increase in swelling from pre-operative to post-operative and compared this between groups. They found that the tranexamic acid group had decreased suprapatellar circumference (considered an index of intraarticular swelling) at one-week postoperatively compared to the control group (TXA group: Mean ​= ​1.6 ​cm, SD ​= ​1.2 ​cm, Control group ​= ​Mean 2.5 ​cm SD ​= ​1.2 ​cm, p=<0.01). Differences were not found at any other timepoint [50]. Further, Huang et al. randomized individuals undergoing TKA to either a combination of topical and IV-administered tranexamic acid (n ​= ​92) or IV application alone (n ​= ​92). Among outcomes related to AMI they measured swelling via knee circumference to calculate a swelling ratio (circumference of operative limb/circumference of contralateral limb) on postoperative days 1, 2, 3, and 4. They found reduced post-operative knee swelling at post-operative days 2, 3, and 4, in the combined topical and IV-administered tranexamic acid compared to IV application alone [51]. Overall, few studies exist that have examined tranexamic acid's impact on knee joint swelling and none that we are aware of have specifically investigated the impact of tranexamic acid on knee extensor strength shortly after TKA as an indirect measure of AMI.

### 2.3 Reducing knee joint swelling

The rationale for targeting post-operative knee joint swelling after TKA to reduce quadriceps AMI is based on the observation that the degree of knee swelling induced by surgery relates to the size of the acute knee-extension strength-loss [4,52,53]. Evidence of causality comes from experimental models of clinical knee swelling where a 60 ​mL intra-articular fluid injection acutely reduced knee-extension strength by 30 ​% on average [26] and as little as 5 ​mL of isotonic saline solution injected into a healthy human knee produces reductions in knee extension strength [29]. Thus, interventions that manipulate knee joint fluid can change indirect measures of quadriceps AMI (knee-extension strength). This effect is likely mediated via increased afferent signaling from knee joint afferents in response to the swelling [6]. However, as introduced earlier, further investigation should explore the relationship between reducing clinical knee swelling and quadriceps activation shortly after TKA.

#### 2.3.1 Intra-articular drains

Intra-articular closed suction drains for TKA are routinely used with the goal of preventing formation of intra-articular haematoma, promoting wound healing, and decreasing associated complications [54]. However, the proposed effects of closed suction drains for TKA are not well supported. A 2016 systematic review with meta-analysis concluded that the use of closed suction drains for TKA offers no improvement in many important outcomes including infection rates, blood loss, wound haematoma, pain, deep venous thrombosis, or range of motion [55], and further research has found similar results [56]. Regarding outcomes influencing AMI, Jennings et al. explored the impact of closed suction drains among individuals undergoing simultaneous bilateral TKA. They randomized 29 patients to receive a closed suction drain in one limb and a sham drain in the other and measured quadriceps strength via handheld dynamometry, quadriceps activation via a clinical quadriceps activation battery, intra-articular knee joint effusion via ultrasound and lower limb swelling via bioelectrical impedance and limb girth. All measures were recorded at day 2, 2 and 6 weeks, and 3-months after surgery. This study found no significant differences between the closed suction drain and sham limbs in any of the outcomes at any timepoint [57]. Thus, based on limited evidence, the use of intra-articular closed suction drains for TKA does not appear to improve post-operative outcomes related to AMI (swelling) or that indirectly or directly measure AMI (strength and activation).

#### 2.3.2 Compression garments

The routine use of thrombo-embolic deterrent (TED) elastic compression stockings after TKA is common. However, It is unclear whether elastic compression stockings reduce the risk of DVT in surgical patients who are also receiving pharmacological prophylaxis [58] and they have been shown to be ineffective for swelling control for individuals after TKA [59]. Conversely, medical grade inelastic compression (≥20 ​mm Hg) applied around a swollen limb decreases the amount of interstitial fluid produced, enhances the efficiency of muscle pumping as the muscles contract against a relatively inelastic barrier, and prevents the backflow of fluid distally [60]. Prior research on inelastic compression in individuals after TKA has been inconclusive regarding its efficacy for swelling control [61]. However, previous studies have been limited in several key aspects. Studies on compression garments for swelling control may be limited by features of the garments including too little compression (e.g., TED stockings) or non-standardized application (e.g., wraps) [59,[62], [63], [64], [65], [66]], application of compression for 48 ​h or less following surgery [59,62,[65], [66], [67], [68], [69]], application of compression locally to the knee only [64,67], and use of professionally applied wraps that the patient cannot remove or hose that is very difficult to apply or remove over a swollen limb [59,[62], [63], [64], [65], [66],^69^]. Finally, some studies have relied on circumferential measurement to measure swelling which may not be accurate or responsive to change depending on the specific measurement [70]. In knees with chronic synovitis, a circumferential measurement (1 ​cm above the patella) correlates with the volume of synovial fluid aspirated from the knee joint [71,72]. However, given that circumferential methods cannot distinguish between edema volume and muscular volume, in the post-TKA setting other methods such as bioimpedance may be considered more precise [73].

An innovative compression garment, that considers the limitations of previous research represents a simple, non-invasive modality that could be used to explore swelling reduction in this population. Carmichael et al. investigated the feasibility and initial efficacy of a multimodal swelling control intervention that included an inelastic adjustable compression garment, manual lymph drainage massage, and a home exercise program compared to a historical control group who received the standard elastic, non-adjustable, TED stocking prescribed in hospital. The multimodal swelling control intervention was initiated on the third or fourth day following TKA, and outcomes were assessed on days 4, 7, 14, 21 and 42 after TKA. The primary outcome measure was lower limb swelling measured by single frequency bio-electrical impedance at two weeks post-operatively. The multimodal swelling intervention group demonstrated less swelling at two weeks postoperatively [effect size 1.28 (95 ​% CI 0.67, 1.88)] and at all other post-operative time points compared to the control group [74]. This suggests that an inelastic compression garment may improve knee swelling after TKA–however, further research is needed given the limitations surrounding study design and small sample size. Future research should explore early application of inelastic compression garments (in the immediate post-operative period) and combine accurate measures of swelling with strength and activation measures such as twitch interpolation to understand the impact of compression garments on AMI.

#### 2.3.3 Athrocentesis

Therapeutic arthrocentesis involves aspirating fluid from the knee joint with the goal of reducing swelling, thus improving pain and function. It has been studied in clinical populations such as knee arthritis with mixed results. Fahrer et al., Geborek et al., and Rice et al., performed arthrocentesis on individuals with chronic swelling due to knee arthritis and demonstrated increases in strength after aspiration of as little as 15 ​mL of intraarticular fluid [47,75,76]. However, Geborek et al. found no change in muscle activity as measured by surface electromyography [47]. Further Jones et al. found no significant increase in activation after arthrocentesis among individuals with knee OA or chronic inflammatory arthritis [77]. It is important to consider that this prior work in arthritis explores the impact of arthrocentesis on chronic swelling, whereas acute swelling induced by surgical trauma such as after TKA may respond differently. Although arthrocentesis is generally regarded as safe, iatrogenic infection is possible [78], therefore this should be weighed against the potential therapeutic benefit. Given mixed results of therapeutic arthrocentesis on chronic swelling, future investigations among TKA populations should intervene early when swelling is in acute stages, as this has not yet been explored.

Overall, based on the profound postoperative swelling that occurs following TKA and limited effective swelling management, there is a critical need for an effective treatment to control swelling after TKA and further investigation on whether swelling control in the immediate post-operative period can mitigate AMI.

### 2.4 Voluntary muscle contractions

As mentioned previously, quadriceps AMI is caused by reduced neural drive of the quadriceps muscle [6]. Conversely, increased neural drive of the quadriceps muscle, happens acutely during voluntary quadriceps muscle contractions [79] and in response to resistance exercise in healthy subjects [80]. A short-period of high-intensity resistance exercise reduces neural inhibition of the trained muscle in healthy subjects [81]. So theoretically, voluntary quadriceps muscle contractions could help reduce quadriceps AMI by increasing quadriceps neural drive.

In an experimental model of clinical knee swelling, one bout of submaximal quadriceps contractions returned knee-extension strength to pre-injection levels (∼30 ​%) [26]. One explanation for this finding is that sub-maximal quadriceps contractions may disperse fluid throughout the joint leading to decreased localized pressure within the joint capsule and decreased mechanoreceptor discharge. Another explanation for this finding is that the joint capsule may become more compliant which would also decrease joint capsule pressure and joint discharge [26,82]. There is some support for this notion based on data from anterior cruciate ligament reconstruction (ACLR) surgery. Among individuals undergoing ACLR, different forms of exercise therapy are associated with significant improvements in quadriceps activation [83].

In the TKA population, Mikkelsen et al. investigated the effect of 10 RM-loaded fatiguing quadriceps muscle contractions on electromyographic (EMG) indicators of neural drive, in patients 4–8 weeks after TKA [84]. In this cross-sectional study, 24 patients performed one set of knee extensions until contraction failure and EMG activity of the quadriceps was measured during the set. They found that quadriceps muscle neural drive increased from 10 to 100 ​% contraction failure [84], indicated by an increase in the surface EMG signal (%EMGmax) and reduced median power frequency of the EMG power spectrum, a pattern that is also seen in healthy subjects [79]. This increase EMG signal is typically coupled with increased excitatory drive to the alpha-motoneuron pool of the contracting muscle as fatigue develops [84,85]. So, implementing fatiguing quadriceps muscle contractions as part of high-intensity rehabilitation exercise after TKA should theoretically help reduce quadriceps AMI and increase knee-extension strength. However, this has not been observed. Bade et al. and Jakobsen et al. conducted two similar RCTs comparing high-intensity rehabilitation exercise – including high-intensity fatiguing quadriceps muscle contractions – compared to usual care rehabilitation exercise without resistance exercise and fatiguing quadriceps muscle contractions [86,87]. Jakobsen et al. randomized individuals to a 7-week supervised progressive strength training program (n ​= ​35) or 7 weeks of supervised physical rehabilitation without progressive strength training (n ​= ​37), early after fast-track TKA. They found that the progressive strength training intervention was not superior in improving functional performance or strength at 2 and 6-months. Further, Bade et al. randomized participants to a high-intensity (n ​= ​84) or low intensity (n ​= ​78) 11-week rehabilitation protocol, 4 days after TKA. The main components of the high-intensity protocol were progressive resistance exercise and rapid progression to weight-bearing and daily activities. They found that high-intensity rehabilitation was not superior to low-intensity in improving functional measures, ROM, or strength at 3-months and 1-year. So currently, there is no clear evidence that high-intensity resistance exercise, including fatiguing quadriceps muscle contractions, can reduce quadriceps AMI over time to a degree where knee-extension strength is impacted substantially after TKA. The lack of effect among these studies is likely attributable to an inability to overcome decreased quadriceps activation and adequately temper post-operative AMI.

Finally, it is also possible that voluntary muscle contractions induce fluid shifts in the clinically swollen knee joint; a mechanism which is different than increasing efferent activation to overcome AMI. In McNair et al.‘s experimental model of clinical knee swelling, one bout of submaximal quadriceps contractions returned knee-extension strength to pre-injection levels [26]. Clinically, this group of authors have observed that use of submaximal contractions in the presence of AMI after TKA can lead to improved ability to perform a straight leg raise. However, this has not been investigated formally in models of clinical knee swelling or after TKA, therefore we see this as an important area for further exploration.

#### 2.4.1 Neuro-muscular electrical stimulation

The use of neuro-muscular electrical stimulation as an adjunct to training improves muscle strength among healthy individuals [88,89], individuals with pathologic muscle weakness [90,91], and individuals undergoing ACLR [92,93]. After TKA, the goal of utilizing neuro-muscular electrical stimulation is to increase neural drive to the quadriceps muscle allowing for greater muscle contraction intensity compared to voluntary efforts alone. This may allow a sufficient training dose to overcome AMI and optimize muscle strengthening [94,95]. After TKA, most older adults can tolerate sufficient current intensities to evoke recommended contractions (between 25 and 50 ​% maximum voluntary isometric contraction) [96,97]. Stevens-Lapsley et al. conducted a randomized controlled trial examining the use of standardized rehabilitation with neuro-muscular electrical stimulation (n ​= ​35) to improve quadriceps muscle strength after TKA delivered early (initiated within 1 week post-operatively) and frequently (daily) compared to standardized rehabilitation alone (n ​= ​31) [38]. The neuro-muscular electrical stimulation group demonstrated significantly greater improvements than the control group in muscle strength, self-reported and performance-based measures at 3.5 weeks and 1-year post-operatively. Improvements were most pronounced at the early timepoint. Quadriceps muscle strength was nearly 30 ​% better in the neuro-muscular electrical stimulation group compared to the control group at 3.5 weeks post-operatively (NMES: mean ​= ​0.93 SD ​= ​0.41 Nm/kg, Control: Mean ​= ​0.66 SD ​= ​0.24 Nm/kg) and this also translated to better functional performance [38]. Kittleson et al. (2013) narrative review of studies that examined neuro-muscular electrical stimulation after TKA (n ​= ​4) found that despite variable results, it appears that neuro-muscular electrical stimulation delivered early and frequently after surgery and at high intensities can help to overcome AMI and attenuate quadriceps strength loss [94]. Overall, neuro-muscular electrical stimulation applied to the quadriceps after TKA represents a promising area for future work to help attenuate quadriceps AMI after TKA.

In summary, future work should seek to explore staging or combining interventions that will support the effectiveness of voluntary muscle contractions. One example of this is early application of an effective inelastic compression garment for swelling control followed by voluntary quadriceps contractions. Another strategy could be combining voluntary contractions with neuro-muscular electrical stimulation to overcome AMI. Future investigation of voluntary quadriceps contractions with neuro-muscular electrical stimulation should seek to measure activation directly with either twitch interpolation or burst superimposition techniques as this has not been explored in the TKA population and could provide more evidence of impact for neuro-muscular electrical stimulation.

### 2.5 Intra-operative surgical techniques

#### 2.5.1 Tourniquets

Torniquets are utilized during TKA with the goals of improved visualization of the surgical field, to minimize blood loss, and to improve cemented implant fixation [98]. The effect of torniquets on lower-extremity muscle function is an area of interest and concern given deleterious effects such as tissue ischemia, edema, and microvascular damage beneath and distal to the cuff [99]. This compressive muscle injury is theorized to modify the muscle afferent discharge and thus potentially contribute to AMI post-TKA [6,100]. Support for this is observed in experimental animal models, where quadriceps force production is reduced after tourniquet compression of the quadriceps [101]. A clinical study investigating tourniquet use during TKA demonstrates decreased quadriceps muscle volume on MRI, and worse self-reported function up to 6-months post-operatively compared to a no-tourniquet cohort [102]. Further, tourniquet use during TKA leads to worse ability to perform a straight-leg-raise at 1- and 3-days post-operative which suggests early quadriceps function may be negatively affected [103]. Given this preliminary evidence, it has been theorized that the use of torniquets may indeed be contributing to AMI and impede muscle function and early post-operative recovery. However, this has not been observed in randomized clinical trials after other major knee surgeries such as ACLR [104] or after TKA [36,100]. Dennis et al. and Harsten et al. conducted two similar RCTs comparing intraoperative torniquet use during TKA and the effects on early knee-extension strength and found no significant differences between groups at early timepoints (48 ​h) [36,100]. Specifically, Harsten et al. randomized individuals undergoing TKA to the use of a tourniquet intraoperatively (n ​= ​32) or no tourniquet (n ​= ​32) and examined the change in knee-extension strength (primary outcome) from pre-surgery to 48 ​h after surgery. They found no statistically significant difference in the loss of knee extension strength between groups (mean difference 1.5 ​N/kg [95 ​% CI, 1.3–1.6]). Dennis et al. explored the impact of tourniquet use among individuals undergoing simultaneous bilateral TKA. They randomized 28 patients (56 lower extremities) to receive a tourniquet on one lower extremity and no tourniquet on the contralateral limb, and measured quadriceps and hamstring strength at two days, 3 weeks, and 3 months after bilateral TKA and quadriceps activation preoperatively and at 3 weeks, and 3 months post-operatively. Interestingly, Dennis et al. did find slightly lower quadriceps strength in the torniquet group at 3-weeks (group difference ​= ​11.27 Nm [95 ​% CI ​= ​2.33–20.20]; p ​= ​0.01) and 3-months after surgery (group difference ​= ​9.48 Nm [95 ​% CI ​= ​0.43–18.54]; p ​= ​0.03). However, no difference in quadriceps strength was observed between groups on post-operative day 2. We suggest the impacts of AMI would be most pronounced at earlier timepoints, thus postoperative day 2 may best represent torniquet effects on AMI. Further, the clinical magnitude of observed differences in quadriceps strength is small at 3-weeks and 3-months after surgery. Overall, there is no clear evidence that the use of a tourniquet contributes to post-operative AMI among individuals undergoing TKA, or that not using a tourniquet may help mitigate indirect measures of AMI after TKA.

#### 2.5.2 Minimally invasive TKA

Minimally invasive TKA, which typically includes a smaller incision and different techniques resulting in less surgical trauma, has emerged over the past decade as a promising alternative to conventional TKA [105]. Minimally invasive TKA is performed with the goals of decreasing pain and improving functional recovery. With less surgical trauma, it seems reasonable to expect that the result is less pain, swelling, and better function as compared to standard TKA, and some clinical studies support this notion in the short-term [106]. Stevens-Lapsley et al. conducted a randomized controlled trial (RCT) comparing minimally invasive TKA to conventional TKA exploring outcomes related to AMI including quadriceps strength (primary outcome), hamstring strength, quadriceps activation via twitch interpolation, functional and patient reported outcomes measured preoperatively and 4,12, 26, and 52 weeks after surgery [37,39]. Interestingly, they found that at four weeks after TKA, the minimally invasive TKA group had significantly greater improvements in hamstring strength, but not quadriceps strength, which did not translate to improved functional performance [37]. Further, no clinically meaningful differences for any outcome measure were observed at any other timepoint [39]. These findings align with further randomized trials in this area [107], suggesting there is no observable advantage of minimally invasive TKA over conventional TKA on direct measures of AMI (quadriceps activation), and indirect measures of AMI (knee-extension strength).

## 3 Limitations

A limitation to consider is that this is a narrative review and despite our best efforts to comprehensively detail relevant literature, we cannot exclude potential selection bias for included studies. Further, this review is limited to interventions that target local and peripheral drivers of AMI shortly after TKA and does not include interventions that target supraspinal sources of AMI.

## 4 Conclusion

In summary, for individuals undergoing TKA, the use of anti-inflammatory medications, tranexamic acid, cryotherapy, intra-articular drains, torniquets, and minimally invasive surgical techniques for TKA appear to have limited benefit in attenuating indirect and direct measures of quadriceps AMI early after surgery. Given mixed results of therapeutic arthrocentesis on chronic swelling, future investigations among TKA populations should intervene early when swelling is in acute stages, as this has not yet been explored. Finally, interventions such as inelastic compression garments, voluntary muscle contractions, and neuro-muscular electrical stimulation show promise in mitigating or circumventing indirect or direct measures of AMI and should continue to be refined and explored.

## Contributions

Laura Churchill contributed to 1) the conception and design of the study, analysis, and interpretation of the data, 2) drafting the article, and 3) final approval the version to be submitted.

Michael John Bade contributed to 1) the conception and design of the study, acquisition of data, 2) revising the article for critically for important intellectual content, and 3) final approval the version to be submitted.

Ryan Koonce contributed to 1) analysis and interpretation of data, 2) revising the article for critically for important intellectual content, and 3) final approval the version to be submitted.

Jennifer Stevens-Lapsley contributed to1) the conception and design of the study, acquisition of data, analysis, and interpretation of data, 2) revising the article for critically for important intellectual content, and 3) final approval the version to be submitted.

Thomas Bandholm contributed to 1) the conception and design of the study, acquisition of data, analysis, and interpretation of data, 2) drafting the article, revising the article for critically for important intellectual content, and 3) final approval the version to be submitted.

## Declaration of funding

Dr. Michael Bade receives funding from the 10.13039/100000002National Institutes of Health (10.13039/100000002NIH), 10.13039/100000133Agency for Healthcare Research and Quality (10.13039/100000133AHRQ), and Veterans Affairs (10.13039/100000738VA).

Dr. Ryan Koonce receives stock or stock options via partial ownership in a patient education company called OrthoSkool.

Dr. Jennifer Stevens-Lapsley receives funding from the 10.13039/100000002National Institutes of Health (10.13039/100000002NIH), 10.13039/100000133Agency for Healthcare Research and Quality (10.13039/100000133AHRQ), and Veterans Affairs (10.13039/100000738VA).

Dr. Thomas Bandholm was supported by a sabbatical research grant from the 10.13039/501100003554Lundbeck Foundation (Grant number R363-2021-307).

## Role of the funding source

No direct role as it pertains to this manuscript.

## Other disclosures

Dr. Thomas Bandholm: I am an exercise physiologist and physical therapist and may have a cognitive exercise bias.

## Declaration of competing interest

Dr. Ryan Koonce receives stock or stock options via partial ownership in a patient education company ‘OrthoSkool’, who played no role in the current study. The other authors have no conflicts.
